# *Trichoderma*-Inoculated Miscanthus Straw Can Replace Peat in Strawberry Cultivation, with Beneficial Effects on Disease Control

**DOI:** 10.3389/fpls.2018.00213

**Published:** 2018-02-21

**Authors:** Jane Debode, Caroline De Tender, Pieter Cremelie, Ana S. Lee, Tina Kyndt, Hilde Muylle, Tom De Swaef, Bart Vandecasteele

**Affiliations:** ^1^Plant Sciences Unit, Flanders Research Institute for Agriculture, Fisheries and Food (ILVO), Merelbeke, Belgium; ^2^Department of Applied Mathematics, Computer Science and Statistics, Ghent University, Ghent, Belgium; ^3^Epigenetics & Defence Research Group, Department Molecular Biotechnology, Ghent University, Ghent, Belgium

**Keywords:** disease, nutrients, peat replacement, plant-microbe interactions, strawberry

## Abstract

Peat based growing media are not ecologically sustainable and often fail to support biological control. Miscanthus straw was (1) tested to partially replace peat; and (2) pre-colonized with a *Trichoderma* strain to increase the biological control capacity of the growing media. In two strawberry pot trials (denoted as experiment I & II), extruded and non-extruded miscanthus straw, with or without pre-colonization with *T. harzianum* T22, was used to partially (20% v/v) replace peat. We tested the performance of each mixture by monitoring strawberry plant development, nutrient content in the leaves and growing media, sensitivity of the fruit to the fungal pathogen *Botrytis cinerea*, rhizosphere community and strawberry defense responses. N immobilization by miscanthus straw reduced strawberry growth and yield in experiment II but not in I. The pre-colonization of the straw with *Trichoderma* increased the post-harvest disease suppressiveness against *B. cinerea* and changed the rhizosphere fungal microbiome in both experiments. In addition, defense-related genes were induced in experiment II. The use of miscanthus straw in growing media will reduce the demand for peat and close resource loops. Successful pre-colonization of this straw with biological control fungi will optimize crop cultivation, requiring fewer pesticide applications, which will benefit the environment and human health.

## Introduction

In horticulture, plants are often cultivated in soilless growing media comprised mainly of peat. Peat has many advantages, e.g., a good pore structure to hold air and water, a low pH adaptable to different crops, a reasonably uniform texture. Peat is easily available and comparatively cheap (Bohlin and Holmberg, 2004), but is not a good medium to harbor beneficial micro-organisms (Hoitink and Boehm, 1999; Krause et al., 2001) because of its high amount of stabilized carbon (C) and therefore low available energy reserves. It does not provide the food base (carbohydrates, chitin, lipids, etc.) for biocontrol agents to grow (Hoitink and Boehm, 1999). Interest in understanding/engineering the biological properties of growing media and the corresponding rhizosphere microbiome is receiving increased attention (De Tender et al., 2016a,b; Grunert et al., 2016).

Peat extraction for horticulture represents 14–20% of peatlands (International Peat Society, 2008). Peat removal threatens sensitive peatland ecosystems, contributes to the reduction and loss of carbon sinks and increases emissions of greenhouse gasses (Kern et al., 2017). The selection of alternative materials for more sustainable growing media is therefore crucial. Alternative materials must have an appropriate physical structure and create a suitable biological and chemical environment for the plant (Barrett et al., 2016).

*Miscanthus x giganteus*, a promising C_4_ perennial biomass crop, is considered a promising candidate bioeconomy crop. It has high yields, low input demand, good environmental performance, multiple biomass use options, and the potential to grow on land considered marginal for food production (Muylle et al., 2015; Lewandowski et al., 2016). Miscanthus straw can be locally sourced and can be an interesting and sustainable peat replacement material. One possible limitation for the use of miscanthus straw in growing media is its interaction with nitrogen (N) (Altland and Locke, 2011; Frangi et al., 2012). N immobilization occurs when organic materials with high C:N ratios, such as straw and other plant fibers, are microbially degraded (Jackson et al., 2009; Vandecasteele et al., 2016), leading to competition with crops for the available N. Miscanthus straw is rich in (hemi)cellulose, which is a source of C for (biocontrol) fungi (Hoitink and Boehm, 1999).

*Trichoderma* is a fungal genus widely known as a biocontrol agent in agriculture (Lorito and Woo, 2015). Its mode of action is both outside and inside the plant. It provides direct mycoparasitic and antimicrobial activity, as well as indirect effects including induced resistance to pathogens and stress and promotion of growth, development and photosynthetic efficiency of the plant (Lorito et al., 2010). Induced resistance—a state of enhanced defensive capacity developed by a plant when appropriately stimulated—is nowadays considered as an interesting strategy to contribute to durable and sustainable crop protection (Borges and Sandalio, 2015).

The aim of the current research was to investigate the potential to replace peat in strawberry cultivation with three forms of miscanthus straw: pure, extruded and extruded plus pre-colonized with the biocontrol strain *Trichoderma harzianum* T22. Our unpublished results revealed that extrusion of plant fibers kills plant pathogens present on the fibers due to the high temperature reached during the process. In addition, we hypothesize that the *Trichoderma* will employ the miscanthus straw as a colonizable medium, allowing its proliferation and actively performing its beneficial functions. Miscanthus can pose a risk for N immobilization and thus N shortage for the crop. In this respect, strawberry was selected for this research due to its high nutrient demand (Lieten and Misotten, 1993). In addition, strawberry is highly sensitive to diseases and has thus also a high pesticide demand (Vervoort et al., 2017). Therefore, it is important to test the efficacy of the biocontrol strain *T. harzianum* T22. To investigate the potential of extruded and *T. harzianum* pre-colonized miscanthus straw as peat replacement and as strategy for increasing disease resistance in growing media, multiple aspects were measured: plant development, plant nutrient content, fruit yield, susceptibility to the fungal pathogen *Botrytis cinerea* (gray mold), chemical parameters of the growing media (including N immobilization), the rhizosphere microbiome and the strawberry defense response. Our results will contribute to provide an overall understanding of the microbiological, chemical and plant health responses in strawberry plants grown in a more sustainable growing medium based on miscanthus straw inoculated with *T. harzianum* T22.

## Materials and methods

### Experimental design

**Figure 1:**
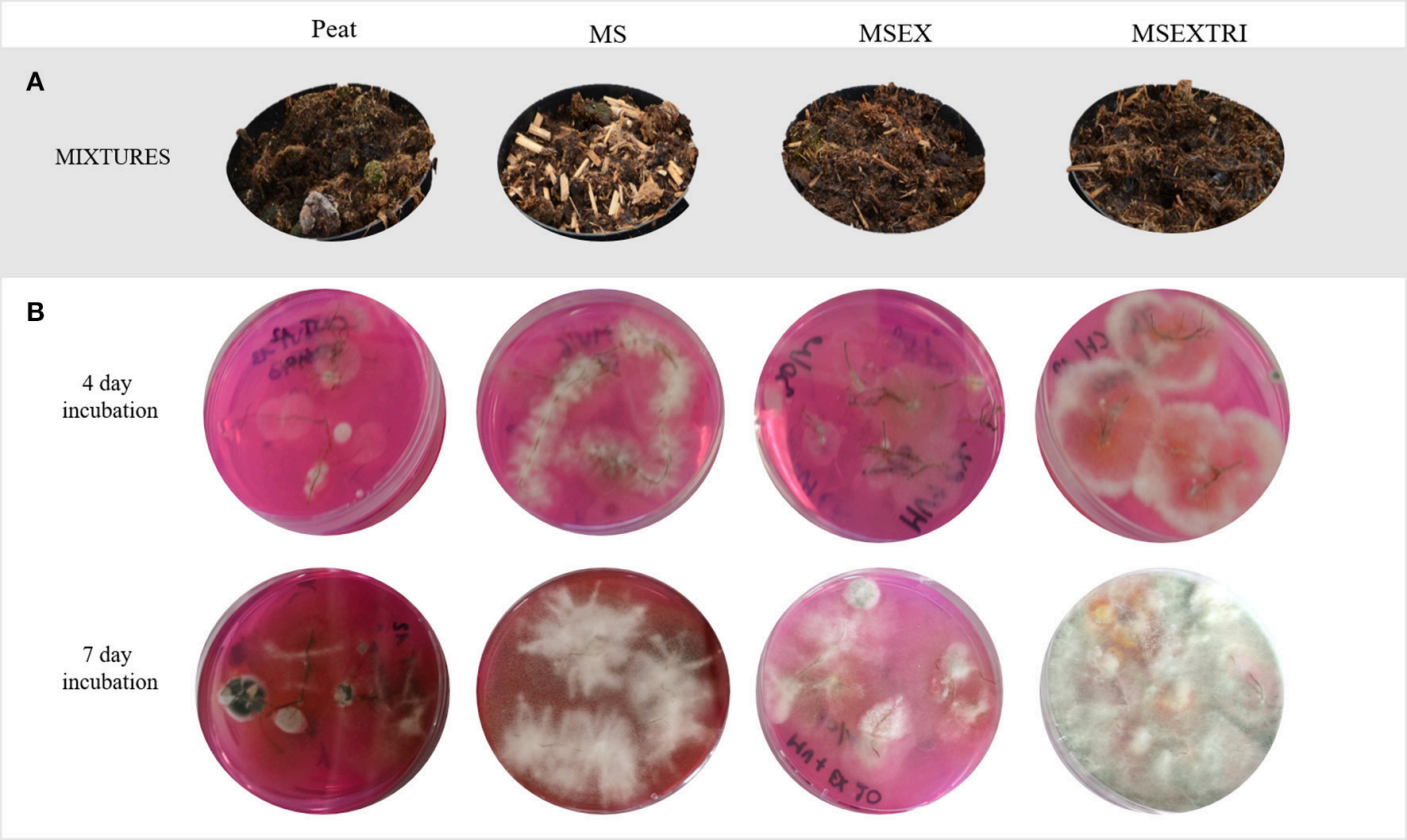
Physical appearance of the different substrate mixtures and their efficiency for *Trichoderma* colonization of the roots. From left to right: peat, control treatment; MS, miscanthus straw; MSEX, miscanthus with extrusion; MSEXTRI, miscanthus straw with extrusion and pre-colonized with *Trichoderma harzianum* T22. **(A)** Peat, amended and not amended with miscanthus treatments (before strawberry planting). **(B)** Strawberry roots of experiment I plated on a semi-selective medium for *Trichoderma* and incubated for 4 days (top) and 7 days (bottom). There were five replicates per treatment.

Two independent experiments were conducted in this study. Experiment I included four mixtures (Figure 1A):

- a fertilized commercial growing medium for strawberry based on 100% white peat [LP108, obtained from Greenyard Horticulture (Ghent, Belgium)], referred to below as peat;
- 80% v/v peat and 20% v/v miscanthus straw (MS);
- 80% v/v peat and 20% v/v extruded miscanthus straw (MSEX), processed with a twin-screw Promeco Extruder System® (PES) at temperatures exceeding 100°C;
- 80% v/v peat and 20% v/v extruded miscanthus straw, pre-colonized by *T. harzianum* T22 (MSEXTRI).

Twenty percentage (v/v) peat replacement by MS was chosen as an optimum balance between reducing the risk for N immobilization and providing sufficient volume of a carrier for biocontrol fungi. The PES twin-screw extruder has short screws that agglomerates the input material combining the effects of pressure and mechanical friction, with a significant temperature increase of the material in the extrusion chamber. The pre-inoculated miscanthus straw was produced under sterile conditions by Mycelia (Nevele, Belgium). In short, *T. harzianum* T22, commercially available as Trianum® (Koppert, the Netherlands), was inoculated as spawn on sterilized grain starting from mother cultures, then mixed with the straw in a straw blender and incubated with the extruded miscanthus straw at room temperature for 11 days for further inoculation.

A complementary experiment (II) was dedicated to: (1) repeat experiment I (mixtures 1–4) and (2) compare pre-colonized extruded miscanthus straw with the direct addition of the *Trichoderma* spores to the growing medium (= commercial use). For the latter, an extra factor (TRIspores) was included, resulting in eight mixtures (Table 1). The same miscanthus straw and extruded miscanthus straw were used as in experiment I, but the extruded straw had a long term storage period and was pasteurized before use. The company “Mycelia” inoculated the pasteurized fibers with *T. harzianum* T22 shortly before use in experiment II. The TRIspores treatment (1 × 10^8^ CFU g^−1^) was applied as for commercial use (750 g m^−3^) by Greenyard.

**Table 1:** Experimental design.

**Mixtures**	**Peat (%)**	**Miscanthus (%)**	**Extruded miscanthus (%)**	***Trichoderma* pre-colonized on the extruded miscanthus**	***Trichoderma* spores added directly**	**Experiment**
Peat	100					I & II
MS	80	20				I & II
MSEX	80		20			I & II
MSEXTRI	80		20	✓		I & II
Peat + TRIspores	100				✓	II
MS + TRIspores	80	20			✓	II
MSEX + TRIspores	80		20		✓	II
MSEXTRI + TRIspores	80		20	✓	✓	II

*Peat replacement is expressed as % (v/v)*.

In both experiments, growing media blends were made and incubated for 1 week at 15°C. After incubation, 25 pots (1.5L) were filled and one strawberry plant (*Fragaria x ananassa* cv. Elsanta) was planted per pot. Additionally, nine pots per substrate mixture were kept without strawberry plants to assess the N immobilization by the growing media; three pots per treatment were sampled at the beginning, midway and at the end of the experiment. All pots were placed for 3 months in the greenhouse and flooded 3–4 times per week until 3.5 cm height (30 min. per event) with an ebb and flood fertigation Quick Valve system containing fertilizer [20N-5P-10K (2 Mg)]. The fertigation water was sampled and analyzed twice for each experiment, i.e., each time the tank was refilled.

### Plant development and fruit susceptibility against gray mold

At the end of the experiment, specific leaf area (SLA) was calculated based on total leaf area (TLA) measured with a high resolution flatbed photo scanner (Perfection V800 Photo, Epson Ltd., Hemel Hempstead, UK) and analyzed using ImageJ, plus the dry weight (DW) of the lamina of all leaves per pot at five pots per mixture (*n* = 5). Furthermore, chlorophyll content was estimated for all leaves of these five plants per mixture using a CCM-200 chlorophyll content meter (Opti-Sciences Inc., Hudson, NH, USA). The output was expressed in Chlorophyll Concentration Index (CCI), defined as the ratio of transmission at 931 to 653 nm through a leaf (Opti-Sciences Inc., USA). Total plant CCI was calculated as a weighted average based on individual leaf area.

Ripe fruits were harvested, counted and weighed at the end of the experiment for all plants for each mixture. Fresh weight (FW) and DW (48 h at 70°C in ventilated oven) of the harvested aboveground biomass was determined. In experiment II, number of stolons and runners per plant and root development (scores 1-3) were also measured at the end of the experiment. For the root development, depending on the number of visible roots (lateral roots and root hairs) on the surface, a 1–3 developmental score was given, with 1 = a few roots; 2 = roots all over the substrate surface; and 3 = substrate surface fully covered with roots.

Harvested fruits were infected with *B. cinerea* isolate 895 (Debode et al., 2013) based on the method of Bhaskara Reddy et al. (2000). Briefly, individual ripe strawberry fruits were inoculated with 20 μl conidial suspension (2 × 10^5^ conidia ml^−1^) and incubated at 11°C in humid conditions. When the first symptoms appeared (about 1 week after inoculation), the strawberries were evaluated daily for 3–4 days and spoiled fruits were discarded each day to avoid secondary infection. The percentage of spoiled fruits per time point was recorded and the area under the disease progress curve (AUDPC) was calculated (Campbell and Madden, 1990).

### Chemical analysis

Total N (Dumas method, ISO 16634-1, Thermo Scientific flash 4000 N analyzer, Massachusetts, United States) and P, Ca, K, Mg (ashing and digestion with 7N HNO_3_ (p.a. 65%) and measured with a Varian VISTA-PRO ICP-OES, Palo Alto, USA) concentrations in the leaves were determined for a composite sample per treatment to assess the limiting nutrients for crop growth. Lignin content in the fibers and leaves was determined using an Ankom220 Fiber Analyzer extraction unit (Ankom, Macedon NY, USA) according to Van Soest et al. (1991).

N immobilization of the pure miscanthus straw and the growing media without strawberry plants was assessed using the protocol described by Vandecasteele et al. (2016) by adding 350 mg N/L fiber or substrate as KNO_3_ (Merck, pro analyse, >99%) followed by 1 week of incubation at 37°C and measuring mineral N concentrations (EN 13652). Based on the difference between the theoretical (initial water-extractable mineral N concentration + added amount of 350 mg N/L material) and actual water-extractable mineral N content after this incubation period, the % N immobilization was calculated. Under 100% N immobilization, all of the 350 mg mineral N/L material would be immobilized. Negative values indicate net N release rather than N immobilization.

Electrical conductivity (EC) (EN 13038) and pH_H_2_O_ (EN 13037) in the fibers and growing media blends were measured in a 1:5 substrate to water (v/v) suspension. Water-extractable C in the peat and fibers was measured in the 1:5 water extract (EN 13652) with ICP-OES. K, Ca, Mg, and P in the growing media blends were extracted (1:5 v/v) in ammonium acetate buffered at pH 4.65 and measured by ICP-OES. Miscanthus straw and growing media were dried for 4 days at 70°C in a ventilated oven, then mechanically ground in a cross beater mill (SK100, Retsch, Haan, Germany). After sample preparation and determination of dry matter (DM) content according to EN 13040, organic matter (OM, EN 13039) content and total N concentration (Dumas method, EN 13654-2, Thermo scientific—flash 4000 analyzer) was determined. The C/N ratio of the growing media was calculated by assuming that C = OM/1.80.

Fertigation water was analyzed for NO_3_, SO_4_, and PO_4_ with a Dionex DX-3000IC ion chromatograph, for NH_4_-N with a Skalar (Skalar Analytical, Breda, The Netherlands) SAN++ flow analyzer and for the other elements with a Varian VISTA-PRO ICP-OES.

### Microbial analysis

#### Colonization of the root endosphere by *Trichoderma*

Survival of *Trichoderma* in the strawberry roots was assessed at the end of each experiment (= 13 weeks of plant growth). Five strawberry roots per treatment were superficially disinfected in 1% NaOCl for 1 min and rinsed 2x in sterile water. Subsequently, the roots were incubated on potato dextrose agar medium amended with 25 mg l^−1^ rose bengal, 100 mg l^−1^ streptomycin, and 300 mg l^−1^ chloramphenicol. This is a semi-selective medium for *Trichoderma* spp. (Elad et al., 1981; Figure 1B).

#### Rhizosphere microbiome

Sequencing of the bacterial and fungal rhizosphere community was done at the end of each experiment. For each growing medium mixture (Table 1), the rhizosphere of five plants was sampled after 13 weeks of plant growth. Rhizosphere sampling was based on the method of Lundberg et al. (2012). Briefly, roots were placed in a sterile 50 ml tube containing 25 ml phosphate buffer. Tubes were vortexed at maximum speed for 15 s, which released most of the rhizosphere soil from the roots and turned the water turbid. The turbid solution was then filtered through a 100 nm nylon mesh cell strainer into a new 50 ml tube to remove plant parts and large sediment particles. The turbid filtrate was further centrifuged for 15 min at 3200 g to form a loose pellet containing fine sediment and microorganisms. Phosphate buffer was removed and the resulting rhizosphere pellets (250 mg) were immediately used for DNA extraction with the PowerSoil DNA isolation kit (Mo Bio, Carlsbad, CA, USA) according to the manufacturer's instructions. DNA was stored at −20°C until further use.

The extracted DNA was used for identifying bacterial (V3-V4 16S rRNA gene) and fungal rhizosphere populations (ITS2) through amplicon sequencing using Illumina technology (Illumina, San Diego, CA, USA). Fragments were amplified and extended with Illumina specific adaptors by using an amplification and dual-index PCR successively (detailed description in Debode et al., 2016). Each PCR step was followed by a PCR product clean-up using the CleanPCR reagent kit (MAGBIO, Gaithersburg, MD, USA). The quality of the final libraries was checked using the Qiaxcel Advanced with the Qiaxcel DNA High Resolution kit (QIAGEN, Germantown, MD, USA) and concentrations were measured using the Quantus double-stranded DNAassay (Promega, Madison, WI, USA). The final barcoded libraries of each sample were diluted to 10 nM and were equally pooled. Resulting libraries were sequenced on an Illumina MiSeq 2x 300 bp paired-end by Macrogen (Seoul, South Korea), using 30% PhiX DNA as spike-in.

De-multiplexing of the amplicon dataset and barcode removal was done by the sequencing provider. The raw sequence data is available in the NCBI Sequence Read Archive under the accession numbers PRJNA398479 and PRJNA398478 for the bacterial and fungal sequences, respectively. The sequence read processing is described in detail in De Tender et al. (2016a,b).

### RNA extraction and gene expression analysis

The RNA material was extracted from strawberry leaves of 2-month-old plants from experiment II as described by Luypaert et al. (2017). The samples underwent DNase treatment (kit DNA-free, cat. Nr. 1906, Ambion, Thermo-Fisher Scientific). The RNA concentration was measured with Nanodrop and Quantus fluorometers. The integrity and quality of the RNA was verified with Experion (Biorad). All the RNA samples were diluted to achieve an equalized concentration of 30 ng/μl. The RNA samples were converted into cDNA with the Tetro cDNA Synthesis Kit (Bioline) following the manufacturer's instructions. cDNA functionality was evaluated through a standard RT-PCR for the reference gene *FaACTIN* (Table S1) and then visualized in an agarose gel (1.5%).

In total, 24 samples (8 treatments × 3 biological replicates) and 10 genes (3 reference genes and 7 defense-related genes, Table S1) were tested. Reference genes were chosen based on data provided by Amil-Ruiz et al. (2013). The target genes are defense-related genes previously reported to respond to pathogen infection in strawberry plants (Table S1). Most of them belong to pathogenesis related (PR) protein families, while Calcium dependent protein kinase (FaCDPK) is involved in Ca-dependent signaling and phenylalanine ammonia lyase (FaPAL) in synthesis of structural and chemical defense molecules through the phenylpropanoid pathway.

The qPCR program was the same for all genes; denaturation (95°C × 2 min), then 40 cycles of denaturation (95°C × 5 s), annealing (58°C × 15 s) and extension (72°C × 10 s) and a final extension of 10 min at 72°C. All qPCR reactions were done in triplicate. To evaluate amplicon specificity, melting peaks were generated and visually inspected at the end of each run.

### Statistical analysis

N immobilization by the pure miscanthus straw (MS, MSEX, and MSEXTRI) was tested in three trials (three replicates each) with two-way ANOVA and Tukey HSD *post-hoc* tests for the factors “Trial” and “Fiber,” without significant effect of the trial on the N immobilization (*p* > 0.05). N immobilization, total N content and C/N ratio in the growing media of the pots without strawberry plants was tested with 3-way ANOVA (factors: experiment, mixture and period) and Tukey HSD *post-hoc* tests. Plant data and foliar concentrations were processed per experiment by one-way ANOVA (experiment I, factor: mixtures) and two-way ANOVA (experiment II, factors: mixture and TRIspores) and Tukey HSD *post-hoc* tests.

OTU tables of the 16S V3-V4 rRNA gene and ITS2 amplicon sequencing were analyzed using the QIIME software package (v1.9.0) (Caporaso et al., 2010b). Taxonomy was assigned with the script “assign_taxonomy.py” using the uclust method considering maximum three database hits, with the silva v119 97% rep set (provided by QIIME) as reference for the bacterial sequences and UNITE v7 (dynamic) for fungal sequences (Caporaso et al., 2010a; Quast et al., 2012; Kõljalg et al., 2013).

For the analysis of the bacterial and fungal microbial community of the rhizosphere, both community diversity and composition were studied. To study community diversity, data was rarefied at 25,000 sequences, both for bacterial and fungal sequences. Based on this rarefied data, the chao1 index, the number of observed OTUs and the Shannon-Wiener diversity index were calculated as an estimation of the community's richness and diversity. To test differences in Chao1 indices, number of observed OTUs and the Shannon-Wiener indices between mixtures, ANOVA analysis was performed followed by a Tukey HSD *post-hoc* test if significant differences were detected. This was done if data normality (tested by the Shapiro Wilk test) and homogeneity of variances (tested by the Bartlett test) were present. In case of heterogeneity of variances, the Kruskall-Wallis test was used. If significant differences were detected by this global test, *post-hoc* tests according to Dunn for pairwise multiple comparison, with Bonferroni correction were applied.

Total community composition differences between groups, in which the different mixtures and repetition of the experiment were indicated as main factors, were analyzed using the multivariate analysis of the specific R package vegan (version 2.3-2) (Oksanen et al., 2010). The dissimilarity matrix, based on the Bray-Curtis dissimilarity index, was calculated from the OTU table as generated by Usearch for bacterial and fungal sequences. Using the betadisper function, the homogeneity of variances was checked on the dissimilarity matrix. Further, the significance of mixture and experiment and their interaction effect was analyzed using PERMANOVA analysis, in which the Bray-Curtis dissimilarity index matrix was used as input.

Second, we assessed differential abundance using likelihood-ratio tests. We tested for (1) the effect of mixture, and (2) the effect of TRIspores addition. The analyses were done upon clustering the bacterial and fungal OTU tables on genus level. An extensive description of the normalization, empirical Bayes estimation and statistical tests performed can be found in De Tender et al. (2016b).

To analyze changes in gene expression between treated samples and the reference sample, we used the relative quantitation technique. For normalization, three internal strawberry reference genes were selected based on the research of Amil-Ruiz et al. (2013). To determine the fold change in gene expression relative to a control or untreated sample, the following formula was used (Livak and Schmittgen, 2001):

2^−ΔΔCt^ (where ΔΔCt = (Ct GI [unknown sample]–Ct GI [reference sample])–(Ct reference genes [unknown sample]–Ct reference genes [reference sample]).

Statistical significance of the differential expression levels was analyzed using a linear model containing mixture and presence of TRIspores as main effect and the interaction term between mixture and TRIspores. Prior to this analysis, data normality and homogeneity of variances was tested by the Shapiro-Wilk test of normality and the Bartlett's test, respectively. All analyses were done in R version (version 3.1.0) (R Core Team, 2015).

## Results

### N immobilization in miscanthus straw and growing media

First, the characteristics of pure peat, pure miscanthus straw (MS) without or with extrusion (EX) and with *Trichoderma* pre-inoculation (TRI) were analyzed. MS, MSEX, MSEXTRI and peat all had high OM contents, high C/N values, pH values between 6.5 and 7.0 and low values for electrical conductivity (Table S2). As the peat based growing medium blend was fertilized, this mixture had a lower C/N ratio than MS, MSEX, and MSEXTRI. N immobilization in the pure miscanthus straw (70 ± 12%) was significantly higher compared to pure extruded miscanthus (40 ± 10%) and *T. harzianum* pre-colonized extruded miscanthus (54 ± 10%). Compared to pure straw, N immobilization in peat (0 ± 16%) was low. MS, MSEX, and MSEXTRI had higher water-extractable C concentrations and lower lignin contents than peat (Table S2).

Second, we tested the effect of Experiment (I vs. II), mixture (Table 1) and sampling period on N immobilization, total N content and C/N ratio, measured in the pots without strawberry plants at three time points. Results are shown in Table S3. There were no interactions between these factors (*p* > 0.05), and the N immobilization was not affected by the mixture tested (*p* > 0.05) nor by the experiment (*p* > 0.05). Therefore, there is no indication of an elevated N immobilization in the growing media with miscanthus used in this study. However, the period had a significant effect on N immobilization, total N content and C/N ratio (*p* < 0.001), with N immobilization being significantly higher at the end of the trial (30%) than during the intermediate sampling (21%), which was significantly higher than the initial situation (4%). There was a clear and significant reduction of the C/N from the initial (67) to intermediate (58) to the end situation (50). Meanwhile, there was a clear and significant increase of the total N content from the initial (0.76%) to intermediate (0.90%) to the end situation (1.01%). These changes in C/N and total N are quite large; we can thus conclude that during the trial important amounts of N are sorbed by the substrates, and that the extent of this sorption was similar for all tested mixtures. At the end of the trials, mineral N concentrations in the growing media of the pots with strawberry plants were below the limit of quantification for all treatments, illustrating that N was either strongly taken up by plants and/or immobilized by the media.

Although large differences in N immobilization were observed between the pure peat vs. MS, MSEX, and MSEXTRI; the mixtures of MS, MSEX, and MSEXTRI with the fertilized peat-based commercial growing medium had lower N immobilization, indicating that blending 20% (v/v) of miscanthus fibers with peat strongly reduces the risk for N immobilization. This was reported before by Vandecasteele et al. (2017).

### Fertigation and nutrient uptake by the plant

All mixtures initially contained suboptimal P, K and mineral N concentrations for plant growth (Table S4), illustrating that the growing media themselves were not a sufficient source for these nutrients for the plants. However, the experiments were run under non-nutrient limiting conditions, since the fertigation water was high in mineral N and other nutrients (Table S5).

#### Experiment I

Although suboptimal N concentrations were measured in leaves (Table 2), there were no significant differences between the treatments for the N concentration in the leaves nor for the total N amount in the leaves (concentration x leaf DM biomass; results not shown). No differences between the treatments were observed for Ca, Mg (optimal range), and P (upper limit of suboptimal range) concentrations while K concentrations for the control treatment were significantly lower than for the other treatments and clearly below the optimal range (Table 2), indicating that K was the limiting element for plant growth only for the peat treatment.

**Table 2:** Nutrient concentrations in the strawberry leaves (dry matter (DM) basis) for several substrates at the end of experiment I and II.

**Factors (TRIspores and mixture)**	**N**	**P**	**K**	**Ca**	**Mg**
	**(%)**	**g/kg DM**			
**EXPERIMENT I**					
**Mixture**					
Peat	1.9 a	1.9 a	7.4 a	10.6 a	5.6 b
MS	1.8 a	2.1 a	13.2 b	9.9 a	4.6 a
MSEX	1.9 a	2.1 a	11.3 ab	10.4 a	4.7 a
MSEXTRI	1.9 a	2.3 a	11.8 ab	10.3 a	5.0 ab
Deficiency	<1.9	<2	<13	<5	<3
Optimal range	2.0-2.8	2.5-4.0	15-25	7.0-17	3.0-5.0
**Statistical significance**					
Mixture	ns	ns	^*^	ns	^*^
**EXPERIMENT II**					
**TRIspores**					
Without	1.7 a	2.2 a	10.0 a	11.7 a	4.8 a
With	1.7 a	2.3 a	10.2 a	11.8 a	4.9 a
**Mixture**					
Peat	1.8 a	2.0 a	6.9 a	13.0 b	5.9 b
MS	1.7 a	2.2 a	12.4 c	11.1 a	4.3 a
MSEX	1.7 a	2.3 a	10.7 b	11.2 a	4.5 a
MSEXTRI	1.7 a	2.4 a	10.5 b	11.7 a	4.7 a
Deficiency	<1.9	<2	<13	<5	<3
Optimal range	2.0-2.8	2.5-4.0	15-25	7.0-17	3.0-5.0
**Statistical significance**					
TRIspores	ns	ns	ns	ns	ns
Mixture	ns	ns	^**^	^**^	^**^
TRIspores x Mixture	^*^	^*^	ns	ns	ns

Different letters in each column indicate significant differences between treatments based on Tukey HSD post-hoc multiple comparison. ns, ^*^, ^**^ indicates non-significant (ns) or significant at the 0.05 (^*^) or 0.001 (^**^) probability level, respectively. Reference for optimal range and deficiency for foliar composition are based on Pritts et al. (2015).

#### Experiment II

The results for the second experiment confirmed the previous observations (Table 2), although the total N content in the leaf biomass was now significantly affected by the substrate mixture, without a significant effect of the direct addition of TRIspores. The total N content in the leaf biomass was significantly higher for the control treatment (385 mg N/plant) than for the three mixtures with MS (206 mg N/plant). This is due to the >30% higher leaf biomass in Experiment II as compared to Experiment I for the control treatment (Table 3), resulting in a >30% increase in the N uptake by strawberry plants in this treatment vs. the other treatments. The lignin content in the leaves was significantly higher for the control treatment (4.9%/DM) than for the other mixtures (3.56–3.95%/DM; no interaction between factors), confirming differences in leaf morphology between treatments.

**Table 3A:** Experiment I. Effect of adding Miscanthus straw (MS) with or without extrusion (EX) and with or without *Trichoderma* pre-inoculation (TRI) on strawberry fruit yield, green biomass yield (DW: dry weight, FW: fresh weight), Total Leaf Area (TLA), Specific Leaf Area (SLA), Chlorophyll Concentration Index (CCI), post harvest *Botrytis cinerea* disease incidence ± standard error (SE) and Area Under Disease Progress Curve (AUDPC).

**Mixtures**	**TLA (cm2)**	**SLA (cm2/g DW)**	**CCI (–)**	**Fruit per plant**	**Weight per plant (g)**		**Post harvest** ***Botrytis cinerea*** **disease incidence**			
				**Number**	**FW**	**DW**	**Time (%)**			**AUDPC (–)**
							**1**	**2**	**3**	
n	5	5	5	25	25	25		45		
Peat	2216 ± 309a	140.4 ± 5.4	26.90 ± 0.55	5.20 ± 1.57	86.99 ± 4.54	29.32 ± 1.73	29.37 ± 6.20	49.86 ± 13.16	91.57 ± 6.11	110.33a
MS	1356 ± 581b	138.5 ± 7.5	24.93 ± 6.75	4.00 ± 1.09	72.17 ± 5.71	25.62 ± 1.61	27.65 ± 5.63	47.64 ± 5.63	90.38 ± 4.29	106.66a
MSEX	1389 ± 460ab	145.9 ± 10.5	25.17 ± 5.94	5.39 ± 1.85	75.60 ± 5.40	27.52 ± 1.66	24.75 ± 4.18	50.79 ± 10.97	82.56 ± 4.87	104.45a
MSEXTRI	1362 ± 246b	145.2 ± 8.4	24.39 ± 4.30	5.52 ± 1.12	72.09 ± 4.80	26.70 ± 1.60	11.9 ± 4.53	34.32 ± 11.32	76.35 ± 10.68	78.45b
**Statistical significance**	^**^	ns	ns	ns	ns	ns	−	−	−	^*^

Only when significant differences between treatments: indicated with different letters (tested with post-hoc Tukey HSD test). ns, ^*^, ^**^ indicates non-significant (ns) or significant at the 0.05 (^*^) or 0.001 (^**^) probability level, respectively.

We conclude that K was the limiting element for plant growth in both experiments only for the peat treatment, while N was (to a lesser extent) the growth limiting element for all treatments (Table 2).

### Plant development and disease susceptibility

#### Experiment I

The final TLA was significantly higher in the peat treatment as compared to MS and MSEXTRI; the MSEX treatment was intermediate. No significant difference between treatments was found for CCI or SLA (Table 3A). CCI values typically range between 10 and 50 for individual leaves, with values increasing as leaves age before senescence (Wood et al., 1993; Tilley, 2015). Values in the present study agreed with these previous results but were averaged over the entire plant based on the CCI values and area of the corresponding leaves. None of the treatments had a significant effect for fruit or plant yield. Fruits grown on strawberry plants in the growing medium with extruded miscanthus straw colonized with *Trichoderma* (MSEXTRI) were significantly less susceptible to gray mold as compared to the other three treatments (presented as AUDPC in Table 3A) as observed during 3 post-harvest evaluation times (Time 1, 2, and 3).

#### Experiment II

There was no significant interaction between the two factors: mixtures and TRIspores (with and without), and no effect of the TRIspores treatment (Table 3B), so data were pooled. The treatments did not have an effect on the number of fruits per plant and the root development. However, all three miscanthus treatments had significantly lower FW and DW of the above ground plant parts (fruit, leaf, and petioles), the number of stolons per plant, the total and SLA and CCI (Table 3B). This observation is in contrast to experiment I which showed no clear effect of MS on the strawberry plant development. There were four fruit rot post-harvest evaluation times, presented in Table 3B as Time 1, 2, 3, & 4. There was significantly more disease in the MSEX treatment as compared to the MSEXTRI treatment, whereas the control (peat) and MS treatment were significantly different from the other treatments in terms of post-harvest disease incidence (Table 3B).

**Table 3B:** Experiment II. Effect of adding *Miscanthus* straw (MS) with or without extrusion (EX), with or without *Trichoderma* pre-inoculation (TRI), and with or without Trianum® spores (TRIspores) on strawberry fruit yield, aboveground vegetative biomass yield (DW: dry weight, FW: fresh weight), Total Leaf Area (TLA), Specific Leaf Area (SLA), total CCI, stolon and root development, post-harvest *Botrytis cinerea* disease incidence ± standard error (SE) and Area Under Disease Progress Curve (AUDPC).

**Factors (TRIspores and mixtures)**	**TLA (cm2)**	**SLA (cm2/g DW)**	**CCI (−)**	**Fruit per plant**		**Weight per plant (g)**		**Stolon per plant**	**Root (1-3)**	**Post harvest** ***Botrytis cinerea*** **disease incidence**				
										**Time (%)**				
				**Number**	**FW (g)**	**FW**	**DW**			**1**	**2**	**3**	**4**	**AUDPC (−)**
**TRISPORES**														
n	20	20	40	100	100	40	40	40	40	224				
Without	2012.60 ± 88.28	140.68 ± 2.24	26.74 ± 4.22	12.40 ± 0.60	68.08 ± 3.56	82.14 ± 3.00	25.06 ± 1.01	4.10 ± 0.23	4.10 ± 0.23	5.36 ± 6.19	20.24 ± 14.25	37.50 ± 19.48	59.52 ± 31.41	90.17
With	2107.86 ± 99.90	139.16 ± 1.96	26.89 ± 4.05	13.16 ± 0.64	68.52 ± 3.64	82.57 ± 3.55	24.63 ± 0.44	4.35 ± 0.25	4.35 ± 0.25	5.36 ± 7.88	19.18 ± 19.45	39.11 ± 21.99	58.29 ± 24.32	90.12
**MIXTURE**														
n	10	10	40	50	50	20	20	20	20	112				
Peat	2680.76 ± 61.72a	138.69 ± 1.91	29.52 ± 3.12a	13.72 ± 0.88	91.52 ± 6.28*a*	105.64 ± 3.71a	32.88 ± 1.23a	5.00 ± 0.30a	2.57 ± 0.14	3.57 ± 6.19	19.05 ± 15.17	36.90 ± 20.96	58.33 ± 26.96	86.90ab
MS	1870.42 ± 75.60b	143.21 ± 1.91	25.96 ± 3.24b	12.32 ± 0.84	59.24 ± 4.16b	72.36 ± 2.62b	21.61 ± 0.91b	3.64 ± 0.27b	2.61 ± 0.11	5.95 ± 7.52	14.29 ± 13.33	33.81 ± 24.58	57.42 ± 22.33	79.78ab
MSEX	1872.72 ± 62.98b	135.43 ± 3.11	25.51 ± 2.68b	12.68 ± 0.88	59.52 ± 4.44b	75.50 ± 2.90b	22.50 ± 0.91b	4.11 ± 0.39b	2.85 ± 0.07	9.52 ± 10.31	33.33 ± 26.81	55.95 ± 18.33	69.05 ± 26.81	128.57a
MSEXTRI	1802.82 ± 52.23b	142.59 ± 3.88	26.20 ± 3.68b	12.44 ± 0.92	63.20 ± 4.88b	76.03 ± 4.30b	22.51 ± 1.18b	4.20 ± 0.35b	2.60 ± 0.12	2.38 ± 4.12	12.18 ± 12.09	26.56 ± 19.07	50.82 ± 35.37	65.34b
**STATISTICAL SIGNIFICANCE**														
TRIspores	ns	ns	ns	ns	ns	ns	ns	ns	ns					ns
Mixture	^**^	ns	^**^	ns	^**^	^**^	^**^	^*^	ns					^*^
TRIspores x Mixture	ns	ns	ns	ns	ns	ns	ns	ns	ns					ns

Only when significant differences between treatments: indicated with different letters (tested with post-hoc Tukey HSD test). ns, ^*^, ^**^ indicates non-significant (ns) or significant at the 0.05 (^*^) or 0.001 (^**^) probability level, respectively.

When comparing the peat treatment of experiment II with that of experiment I, higher above ground vegetative biomass in experiment II is illustrated by clearly higher values of TLA, FW, and DW biomass per plant (= leaves + petioles) and fruit yield.

### Microbial responses

#### Root colonization by trichoderma using plating

The white mycelium detected on the semi-selective media after 4 days of incubation in the MSEXTRI treatment, turned greenish after 7 days of incubation (Figure 1B). Moreover, only in the MSEXTRI treatment, typical *Trichoderma* conidiophores and conidia were detected under the microscope. Based on this morphology, it was concluded that in both experiments only the strawberry roots of the MSEXTRI treatment were consistently and fully colonized by *Trichoderma* spp.

#### Rhizosphere microbiome using amplicon sequencing

The strawberry rhizosphere community contained around 2100 and 1450 bacterial OTUs for experiment I and II, respectively, and was estimated to have a similar bacterial richness (Chao1) and diversity (Shannon-Wiener) for all mixtures (Table 4). The fungal community of the strawberry rhizosphere grown in extruded miscanthus pre-colonized with *T. harzianum* was less diverse, however, and contained fewer unique OTUs compared to the other treatments. Conversely, this effect was not observed in experiment II (Table 4), where no statistical differences were observed in richness and diversity measurements and estimates. The reason for this difference in outcome between the two experiments is unclear. The same miscanthus straw was used for both experiments, but as the extruded straw had a long term storage period before the start of experiment II, MSEX was pasteurized before use and this may have affected its colonization by *T. harzianum* and other micro-organisms. Moreover, the strawberry transplants used in experiment II were harvested one year later than in experiment I and had a better growth, which may also have affected the response of the microbial community to the treatments.

**Table 4:** Richness measurement (observed OTUs) and richness and diversity estimates (Chao1 and Shannon-Wiener diversity index) of the bacterial and fungal community of the strawberry rhizosphere, grown in different substrate mixtures.

**MIXTURE**	**BACTERIA**			**FUNGI**		
	**Observed OTUs**	**Chao1**	**Shannon**	**Observed OTUs**	**Chao1**	**Shannon**
**EXPERIMENT I**						
Peat	2106 ± 253(a)	2979 ± 385(a)	8.83 ± 0.37(a)	686 ± 52(a)	813 ± 76(a)	6.54 ± 0.33(a)
MS	2206 ± 174(a)	3096 ± 173(a)	9.16 ± 0.40(a)	587 ± 80(ab)	783 ± 112(ab)	4.79 ± 0.58(b)
MSEX	2083 ± 182(a)	2890 ± 189(a)	8.90 ± 0.38(a)	642 ± 84(a)	812 ± 116(a)	5.41 ± 0.25(b)
MSEXTRI	2194 ± 115(a)	3012 ± 179(a)	9.19 ± 0.14(a)	529 ± 33(b)	684 ± 52(b)	3.72 ± 0.22(*c*)
**EXPERIMENT II**						
Peat	1451 ± 697(a)	2122 ± 965(a)	7.39 ± 1.36(a)	662 ± 85(a)	866 ± 106(a)	5.86 ± 0.77(a)
MS	1292 ± 534(a)	1982 ± 724(a)	6.84 ± 1.24(a)	658 ± 14(a)	864 ± 36(a)	5.53 ± 0.15(a)
MSEX	1770 ± 444(a)	2584 ± 545(a)	8.07 ± 1.16(a)	731 ± 52(a)	1001 ± 73(a)	5.53 ± 0.28(a)
MSEXTRI	1115 ± 141(a)	1743 ± 210(a)	6.59 ± 0.53(a)	637 ± 65(a)	862 ± 117(a)	5.16 ± 0.29(a)
Peat + TRIspores	1618 ± 447(a)	2413 ± 582(a)	7.68 ± 1.11(a)	660 ± 91(a)	888 ± 118(a)	5.48 ± 0.40(a)
MSEX + TRIspores	1305 ± 396(a)	2004 ± 521(a)	7.00 ± 0.84(a)	733 ± 57(a)	1018 ± 88(a)	5.55 ± 0.32(a)

*Values represent means ± standard deviation, based on 5 replicates per mixture (Miscanthus straw without (MS) or with extrusion (MSEX), and with Trichoderma pre-colonization (TRI), TRIspores: with Trianum® spores). Different letters in each column indicate significant differences between treatments*.

**Figure 2:**
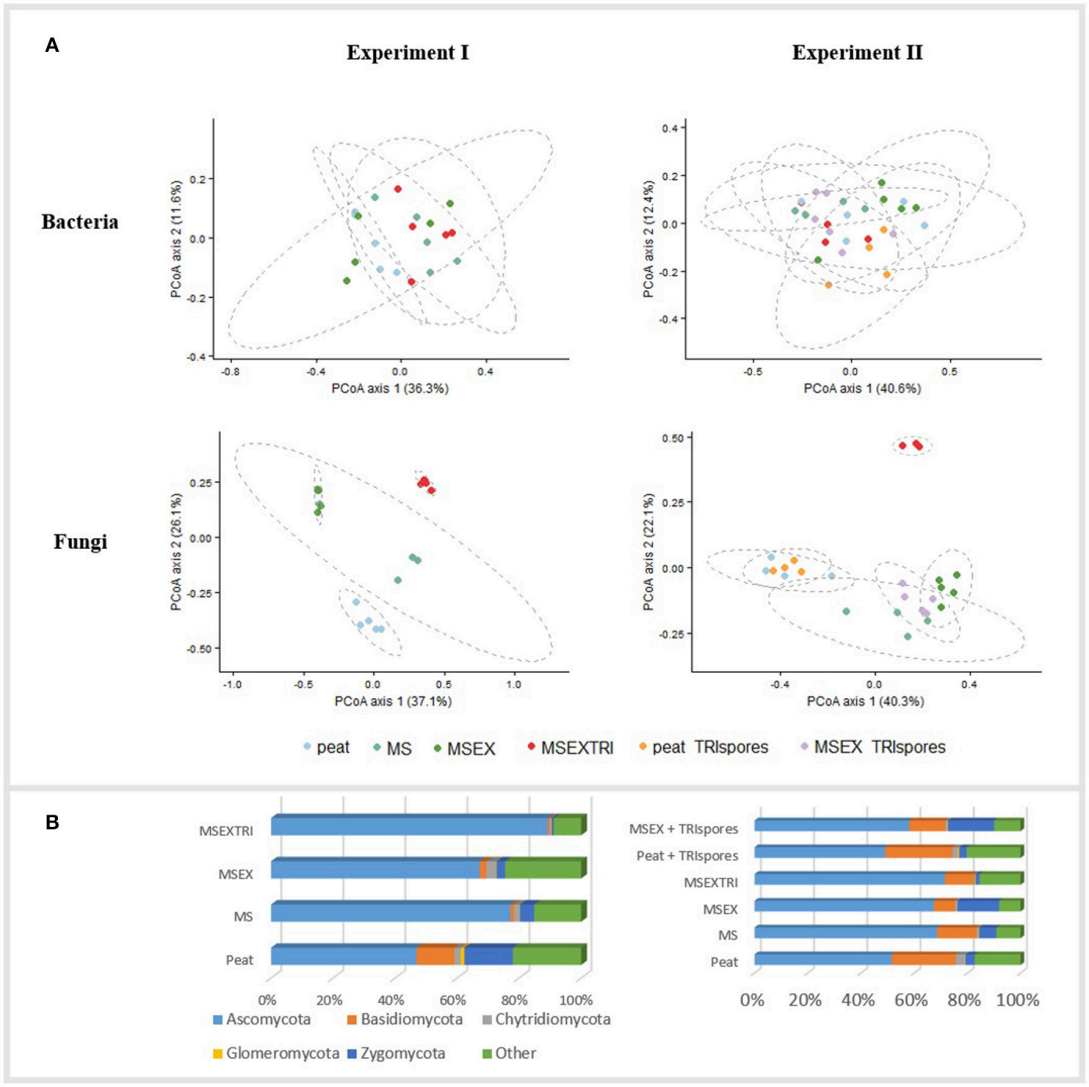
Community composition of the strawberry rhizosphere microbiome for the different mixtures. Peat, control treatment; MS, miscanthus straw; MSEX, miscanthus with extrusion; MSEXTRI, miscanthus straw with extrusion and pre-colonized with *Trichoderma harzianum* T22, and TRIspores: Trianum® spores added directly to the growing medium. **(A)** Principal Coordinate analysis (PCoA) profiles of pairwise community dissimilarity (Bray-Curtis) indices of the V3-V4 region of 16S rRNA genes (bacteria, top figure) and ITS2 sequencing data (fungi, bottom). Ellipses represent the 95% confidence intervals. PCoA plots are given for both Experiment I (left) and Experiment II (right) separately; **(B)** Representation of the fungal phyla in mean relative abundance (%) (*n* = 5), both for Experiment I (left) and Experiment II (right). Sequences which could not be classified on phyla level or to the kingdom fungi are represented in the group Other.

Statistical analysis of the two independent experiments was done individually, both for bacterial and fungal sequences. The bacterial community of the strawberry rhizosphere showed no dissimilarities in composition between mixtures (PERMANOVA, *p* > 0.05; Figure 2A). In contrast, the fungal community structure was strongly influenced by the type of mixture, both for experiment I (PERMANOVA, *p* < 0.01) and experiment II (PERMANOVA, *p* < 0.001) as illustrated by the PCoA plots in Figure 2A. These plots illustrate an effect of the addition of miscanthus and the pre-colonization with *Trichoderma*, but no additional effect of the presence of *Trichoderma* spores (TRIspores) in the growing medium. The differences in the fungal community structure between mixtures is due to a change in relative abundance between at least 2 treatments of 52 genera in experiment I, and 40 genera for experiment II (Table S6), 33 of which are shared between the experiments. These changes are specifically due to differences in Ascomycota members (Table S6, Figure 2B). One genus, *Humicola* sp., increased significantly in relative abundance in MSEXTRI compared to the other mixtures as observed in both experiments (Table S6; Table 5). Other highly abundant genera that increased in relative abundance (particularly in the MS treatment) are linked with (ligno)cellulose degradation, such as *Chaetomium* and *Sporothrix* (Table 5). Several genera (e.g., *Cladosporium, Pilidium*, and *Ilyonectria*) decreased in relative abundance in the mixtures with miscanthus fibers compared with peat containing potentially plant-pathogenic species (Table 5). Remarkably, *Trichoderma* was only present in low abundances (≤0.25%) in the rhizosphere of all treatments, including in the treatments to which it was added (Table S6).

**Table 5:** Description of possible plant- and/or soil-linked functions of statistical significant fungal genera in mixtures compared to peat whose mean relative abundance is at least 1% in at least one mixture type.

		**Experiment I**					**Experiment II**					
**Phylum**	**Genus**	**Peat**	**MS**	**MSEX**	**MSEXTRI**	**Peat**	**MS**	**MSEX**	**MSEXTRI**	**Peat + TRIspores**	**MSEX + TRIspores**	**Possible function(s) (Reference)**
Ascomycota	*Chaetomium*	0.22 ± 0.12	**6.0x**	0.3x	1.6x	0.15 ± 0.05	**5.1x**	0.7x	0.8x	0.8x	1.0x	Cellulosic substrate degradation (Longoni et al., 2012)
	*Cladosporium*	5.23 ± 1.21	0.1x	0.1x	0.1x	1.55 ± 0.79	0.2x	0.1x	0.3x	0.5x	0.1x	Some plant pathogens, endophytic organisms (Bensch et al., 2012)
	*Geomyces*	0.03 ± 0.01	**2.0x**	**19.0x**	<0.1x	1.04 ± 0.56	0.5x	0.3x	0.4x	0.9x	0.4x	/
	*Humicola*	4.07 ± 1.17	**3.6x**	0.3x	**13.3x**	2.53 ± 0.89	**11.4x**	**15.3x**	**18.8x**	2.8x	**12.0x**	Lignocellulose degradation, induced resistance (Ko et al., 2011; Yang et al., 2014)
	*Ilyonectria*	1.15 ± 0.64	*<0.1x*	0.1x	*<0.1x*	0.11 ± 0.05	0.4x	*<0.1x*	0.1x	0.4x	0.1x	Root rot pathogens of herbaceous plants (Cabral et al., 2012)
	*Lecanicillium*	1.12 ± 0.23	0.1x	0.2x	0.1x	0.53 ± 0.24	0.1x	0.2x	0.3x	1.5x	0.3x	PGP, chitin degradation, biocontrol and induced resistance (Goettel et al., 2008)
	*Meliniomyces*	0.11 ± 0.08	0.1x	0.2x	0.1x	1.02 ± 0.29	0.1x	0.1x	0.2x	0.2x	0.1x	Root-associated fungi (Hambleton and Sigler, 2005)
	Ophiostoma^**^	0.13 ± 0.08	0.2x	0.1x	0.6x	2.57 ± 0.67	0.9x	0.5x	1.3x	1.1x	1.0x	Wood fungi (Linnakoski et al., 2010)
	Paraphaeosphaeria^*^	0.02 ± 0.02	8.5x	2.0x	1.0x	0.05 ± 0.02	**65.8x**	**16.0x**	**10.8x**	3.2x	**19.4x**	Plant-associated fungi producing metabolites (Paranagama et al., 2007)
	Pilidium^*^	0.00 ± 0.00	NA	NA	NA	3.00 ± 1.08	0.6x	0.1x	0.5x	0.8x	0.3x	Strawberry pathogen (Debode et al., 2011; Karimi et al., 2017)
	*Podospora*	0.02 ± 0.01	**28.5x**	3.0x	14.5x	0.07 ± 0.04	**10.7x**	**30.6x**	**7.1x**	0.1x	**7.9x**	/
	*Pseudogymnoascus*	0.15 ± 0.03	0.1x	0.8x	0.1x	0.59 ± 0.16	1.8x	1.3x	0.4x	1.2x	1.3x	Biocontrol (Tagawa et al., 2010)
	*Sporothrix*	0.15 ± 0.03	**6.1x**	**7.9x**	**4.2x**	0.14 ± 0.02	**6.1x**	**6.1x**	**6.6x**	3.8x	**12.7x**	Saprophytic species in soil (Rodrigues et al., 2016)
	Tetracladium^**^	1.38 ± 0.90	<0.1x	<0.1x	<0.1x	0.00 ± 0.00	0.6x	0.8x	1.3x	3.7x	0.5x	/
	Zopfiella^**^	0.52 ± 0.18	3.1x	**16.7x**	3.3x	0.09 ± 0.05	0.6x	2.1x	1.1x	0.4x	0.4x	/
Basidio-mycota	*Cryptococcus*	1.69 ± 0.24	0.1x	0.3x	0.1x	5.88 ± 1.61	0.4x	0.3x	0.3x	0.5x	0.3x	Biocontrol & epiphytic fungi (Sharma et al., 2009; Debode et al., 2013)
	*Trichosporon*	3.63 ± 1.85	0.1x	0.1x	*<0.1x*	9.63 ± 2.20	1.2x	0.6x	**1.1x**	**1.8x**	1.1x	/
Zygomycota	*Mortierella*	14.61 ± 3.65	0.3x	0.1x	<0.1x	2.90 ± 0.77	**2.0x**	**5.0x**	0.5x	0.8x	**5.8x**	Chitin degradation, biocontrol (Tagawa et al., 2010)
	*Umbelopsis*	0.96 ± 0.28	0.2x	0.1x	0.1x	0.42 ± 0.09	0.2x	0.5x	0.2x	0.6x	0.2x	/

*The peat column indicates the mean relative abundance (%) ± standard error of the strawberry rhizosphere genera grown in peat. The other mixtures indicate the increase or decrease in mean relative abundance as compared to the peat mixture. Statistical significant increases compared with the peat substrate are indicated in **bold**, significant decreases are underlined,/ means no possible specific functions reported in literature*.

* Genus showed no significant differences between mixtures in Experiment I

** Genus showed no significant differences between mixtures in Experiment II

### Strawberry defense response

**Figure 3:**
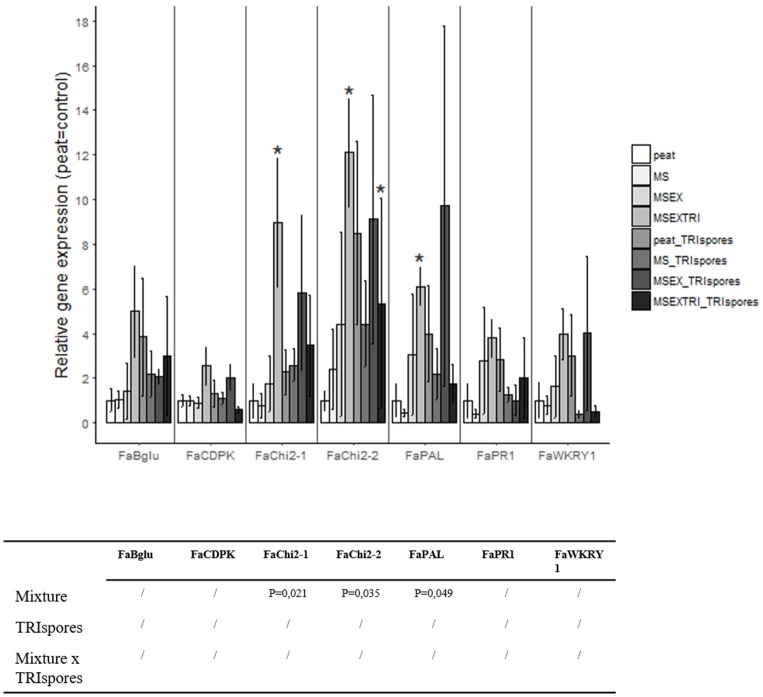
Relative gene expression of seven defense genes in strawberry based on Experiment II. Expression levels are expressed in fold changes, by which the peat treatment is used as control treatment (gene expression is set at 1). Statistical significant differences compared to the control treatment are indicated with an asterisk.

A gene expression analysis using qRT-PCR revealed that strawberry plants grown in treatments where *Trichoderma* was inoculated have a higher transcript level for several plant defense-related genes in comparison to the 100% peat control substrate. Specifically, a significantly higher expression of genes encoding chitinases (FaChi2-1, FaChi2-2) and phenylalanine ammonia lyase (FaPAL) was detected in the strawberry plants grown in the growing medium containing extruded miscanthus pre-colonized with *T. harzianum* (Figure 3). Although not statistically significant in most cases, an induced expression of these three genes was also observed in the treatments where *Trichoderma* spores were directly added to the extruded miscanthus containing growing medium (Figure 3).

## Discussion

Peat use in horticultural growing media is sharply critized because of its ecological footprint, thus alternatives are urgently needed (Barrett et al., 2016). The use of miscanthus straw to replace peat in growing media has been tested by Altland and Locke (2011) and Frangi et al. (2012), where both studies acknowledge the risk for N immobilization. Increasing the dose of N fertilization (Jackson et al., 2009) or improving the accessibility of the fertilizer for the plant roots (Altland and Locke, 2011) may thus alleviate the risk of N immobilization. Increasing the dose of N fertilization is still the most applied and cost-effective method to overcome the negative effects of N immobilization. In the present study, the effect of N immobilization was tested in a system with high N input by fertigation. Although the pure miscanthus straw showed high N immobilization in comparison with peat, measurements in the growing media did not show differences between the treatments. For all treatments, the N immobilization increased and the C/N ratio decreased due to N drawdown during the cultivation, indicating increasing microbial activity in the growing media during cultivation, the underlying process for N immobilization (Jackson et al., 2009; Vandecasteele et al., 2016) and/or higher microbial biomass. This is a topic for future research, by including assessing microbial activity/ microbial biomass during crop cultivation. Foliar analyses in Table 2 illustrate that Ca and Mg were in the optimal range for strawberry, so these elements were not limiting the plant growth in both of the trials. For Ca this is confirmed by analyses of the initial growing media (Table S4). For both experiments, K seemed to be the limiting element for plant growth for the peat treatment. In addition, suboptimal N concentrations were limited for plant growth for all mixtures, without significant differences between mixtures. The uptake of N was higher in experiment II, however, as shown by a higher aboveground plant biomass and a significantly higher total N content in the leaf biomass in the peat treatment as compared to Experiment I. This overall higher crop N uptake in Experiment II resulted in a more limiting effect of N on plant growth, reducing yields by 35% in Experiment II for the treatments with miscanthus straw.

The addition of miscanthus straw to the growing medium altered the fungal rhizosphere community of strawberry, leading the Ascomycota to become predominant. Ascomycota are the major decomposers in acidic, peat-based soils (Thormann et al., 2002). We hypothesize that miscanthus is a more appropriate carbon source than peat for decomposer fungi, such as *Chaetomium* and *Humicola* sp., providing them with an advantage to dominate over other phyla of saprophytic fungi. This is illustrated by the higher water-extractable C concentrations and the lower lignin contents for MS, MSEX and MSEXTRI than for peat (Table S2). It is probable that fungi specialized in decomposing cellulose, hemicellulose and lignin substrates have a faster proliferation when miscanthus was added; in fact, fungi are considered to be the greatest decomposers due to their high efficiency (Sánchez, 2009). Depending on the straw treatment (non-extruded, extruded, or inoculated with *T. harzianum*), the relative proportion of saprophytic fungi in each treatment was different.

To increase disease suppressiveness, the peat-replacing extruded miscanthus straw was pre-colonized by *T. harzianum* T22, and this colonization appeared to be effective in controlling the fungal pathogen *B. cinerea* on the strawberry fruits as compared to the un-inoculated extruded miscanthus straw. The underlying mechanism could be related to an enhanced defensive capacity of the plant induced by *T. harzianum* colonization of the roots, in combination with the altered fungal microbiome on the roots. The first was shown by plating on a semi-selective medium for *Trichoderma*. However, in further research more throughout examination of the *Trichoderma* on the roots is necessary to confirm whether it is the T22 strain that is present (now only determined up to genus level) and to quantify its presence in the roots. This can be done with the strain-specific qPCR published by Horn et al. (2016). The reshaping of the rhizosphere microbiome was shown by amplicon sequencing and our results are in accordance to Xiong et al. (2017) who showed that *Trichoderma* introduced in a bio-fertilizer treatment induced suppressiveness by changes in the soil microbiome rather than direct pathogen inhibition.

We showed the up-regulation of defense related genes in the leaves upon *T. harzianum* treatment. Induced resistance is a state of enhanced defensive capacity developed by a plant when appropriately stimulated. In the case where this phenomenon is caused by root colonization by one or more beneficial micro-organism(s) the term induced systemic resistance is used (ISR). ISR by *T. harzianum* has previously been shown for other crops such as tomato (De Meyer et al., 1998; Harel et al., 2014). The enhanced expression of the defense genes in strawberry leaves of plants colonized with *T. harzianum* was only present when miscanthus straw was pre-colonized with *T. harzianum*, whereas the direct addition of *T. harzianum* spores to mixes was not effective. Similarly, Krause et al. (2001) showed that potting mixes prepared with peat do not lead to ISR in plants, but amendment of such media with lignocellulosic compounds such as composted pine bark increases efficacy of biocontrol agents and leads to systemic resistance. Whether the induced defense is also present in fruits, and if the defense response is further enhanced upon infection (so-called “priming”) remains to be further studied.

Surprisingly, the relative abundance of *Trichoderma* sp. in the rhizosphere community was low for all mixtures (<1%), whereas the relative abundance of the fungal *Humicola* spp. increased significantly, especially when miscanthus was pre-colonized with *T. harzianum*. Little is known about the genus *Humicola*. Chung and Hoitink (1990) showed an interaction between *Trichoderma* and *Humicola* in compost and Harman et al. (2004) showed that *Trichoderma* spp. can parasitize other fungi. Although further experiments are needed to confirm this view, we hypothesize that *Humicola* spp. are resistant to mycoparasitism by *Trichoderma* and therefore can rapidly colonize the empty niche created by *Trichoderma-*induced fungal death in the rhizosphere. Interestingly, the lignocellulose degrading genus *Humicola* is also known to be involved in disease suppression (Ko et al., 2011; Yang et al., 2014; Xu et al., 2016), which could further explain the increased expression of genes encoding anti-fungal enzymes like chitinase 1 and 2 and phenylalanine ammonia lyase in the strawberry leaves.

To conclude, this study shows that miscanthus straw can partially replace peat in growing media when enough N is supplied during the cultivation in order to compensate for N immobilization in miscanthus straw. In addition, we showed that miscanthus straw is a good carrier for the biological control agent *T. harzianum* in order to increase the disease suppressiveness of the growing media.

## Author contributions

BV: supervised the project; BV, JD, and HM: were involved in the design and the supervision of the experiments; JD and PC: conducted the strawberry plant experiments; BV and PC the chemical analysis of the plant, water, and growing media; CDT and AL: the amplicon sequencing; CDT: the bio-informatics of the NGS data and the statistical analysis of the data; TK, HM, and AL: the strawberry defense response analysis and TDS the plant development analysis. JD, BV, and CDT: wrote the first draft and finalized the manuscript. All authors contributed to the writing of the manuscript and approved submission.

### Conflict of interest statement

The authors declare that the research was conducted in the absence of any commercial or financial relationships that could be construed as a potential conflict of interest.
