# Supplementary material for: Trichoderma-Inoculated Miscanthus Straw Can Replace Peat in Strawberry Cultivation, with Beneficial Effects on Disease Control
Source: Front Plant Sci. 2018 Feb 21;9:213. doi: 10.3389/fpls.2018.00213 (PMC5826379; doi:10.3389/fpls.2018.00213)
Supplement: Supplementary file 1 [file Table1.docx]

**Table S1. Primers used in this study for expression of RT-qPCR defense-related genes previously reported in strawberry. We used 7 target genes to study gene expression related to the strawberry plant defense response and 3 reference genes (ref) for data normalization.**

| **Gene** | **Gene description related to ISR** | **function** | **Primers Sequence (Forward (F)/Reverse(R))** | **Literature reference** |
| --- | --- | --- | --- | --- |
| ***FaRIB413*** | RNA interspacer (16S-23S) region | ref | **F** ACCGTTGATTCGCACAATTGGTCATCG  **R** TACTGCGGGTCGGCAATCGGACG | Amil Ruiz *et al*., 2013 |
| ***FaACTIN*** | Actin | ref | **F** GGGCCAGAAAGATGCTTATGTCGG  **R** GGGCAACACGAAGCTCATTGTAGAAG | Amil Ruiz *et al*., 2013 |
| ***FaEF1a*** | Elongation factor 1-alpha | ref | **F** TGGATTTGAGGGTGACAACATGA  **R** GTATACATCCTGAAGTGGTAGACGGAGG | Amil Ruiz *et al*., 2013 |
| ***FaBglu*** | β-1,3-Glucanase | PR2 | **F** TATGGACGAAACGGTGACAA  **R** AGGGTTGCACATTTTTCTGG | Landi *et al*., 2014 |
| ***FaCDPK*** | Calcium-dependent protein kinase | biotic stress signalling, activated by calcium | **F** TCCGTTTTGAAGAACCCAAC  **R** CCGTCCTCAGTTTCTGCTTC | Landi *et al*., 2014 |
| ***FaChi2-1*** | Chitinase gene | PR3 | **F** TCGGCACCACCGGAAGT  **R** TGGGAGATCTGAGCAAGAAATG | Khan & Shih, 2004 |
| ***FaChi2-2*** | Chitinase gene | PR3 | **F** GGTCAAACCTCTCACGAAACCA  **R** ATCCCCAAGCATAAGGACCAT | Khan & Shih, 2004 |
| ***FaPAL*** | Phenylalanine ammonia-lyase | phenylpropanoid pathway | **F** GATTTGAGGCATTTGGAGGA  **R** CTTGCCTTAGCCTTTGCATC | Landi *et al*., 2014 |
| ***FaPR1*** | Pathogenesis-related protein 1 | PR1 | **F** ACATGGATGCCAATCTAGC  **R** CCACAGGTTCACAGCAGATG | Pombo *et al*., 2011 |
| ***FaWRKY1*** | WRKY75 like transcription factor | transcription factor induced by SA, ABA, wounding and infection | **F** ACAGCATAAGATTAGGGATGAAGAAGGGAG  **R** GCTTCTTCACATTGCAACCCTGATGCGTG | Amil Ruiz *et al*., 2013 |

**Amil-Ruiz F, Garrido-Gala J, Blanco-Portales R, Folta KM, Muñoz-Blanco J, Caballero JL. 2013**. Identification and validation of reference genes for transcript normalization in strawberry (Fragaria x ananassa) defense responses. *PLoSONE* 8: e70603.

**Khan AA, Shih DS. 2004**. Molecular cloning, characterization, and expression analysis of two class II chitinase genes from the strawberry plant. *Plant Science* 166: 753–762

**Landi L, Feliziani E, Romanazzi, G. 2014.** Expression of defense genes in strawberry fruits treated with different resistance inducers*.* *Journal of Agricultural and Food Chemistry* 62: 3047−3056.

**Pombo MA, Rosli HG, Martínez GA, Civello, P.M. 2011.** UV-C treatment affects the expression and activity of defence genes in strawberry fruit *(Fragaria x ananassa, Duch.). Postharvest Biology & Technology* 59*:* 94–102*.*
