# Supplementary material for: Trichoderma-Inoculated Miscanthus Straw Can Replace Peat in Strawberry Cultivation, with Beneficial Effects on Disease Control
Source: Front Plant Sci. 2018 Feb 21;9:213. doi: 10.3389/fpls.2018.00213 (PMC5826379; doi:10.3389/fpls.2018.00213)
Supplement: Supplementary file 2 [file Table2.docx]

**Table S2. Characteristics of the pure miscanthus straw without (MS) or with extrusion (MSEX), and with *Trichoderma* pre-inoculation (TRI), and peat applied in the trials (OM: organic matter, DM: dry matter, Nmin: mineral N, C- H_2_O: waterextractable C, N immob.: N immobilization)**

| **Pure fiber** | **OM** | **Total N** | **C/N** | **DM** | **pH-H_2_O** | **EC** | **Nmin** | **C- H_2_O** | **Lignin** | **N immob.** |
| --- | --- | --- | --- | --- | --- | --- | --- | --- | --- | --- |
|  | **%/DM** | **%/DM** |  | **%/fresh** | **-** | **µS/cm** | **mg/L growing medium** | **mg/L growing medium** | **%/DM** | **%** |
| Peat | 94.3 | 0.9 | 56.2 | 36.1 | 6.6 | 123 | 35.7 | 166 | 20.9 | 0 |
| MS fiber | 98.1 | 0.3 | 191.9 | 86.2 | 6.9 | 87 | < 5.0 | 514 | 10.9 | 70 |
| MSEX fiber | 97.7 | 0.3 | 171.2 | 53.3 | 7.3 | 116 | < 5.0 | 751 | 12.2 | 40 |
| MSEXTRI fiber | 97.2 | 0.6 | 93.7 | 25.7 | 6.9 | 259 | 34.0 | 1733 | 15.8 | 54 |
