# Supplementary material for: Trichoderma-Inoculated Miscanthus Straw Can Replace Peat in Strawberry Cultivation, with Beneficial Effects on Disease Control
Source: Front Plant Sci. 2018 Feb 21;9:213. doi: 10.3389/fpls.2018.00213 (PMC5826379; doi:10.3389/fpls.2018.00213)
Supplement: Supplementary file 3 [file Table3.DOCX]

**Table S3. N immobilization (N immob), organic matter (OM), pH, total N and C/N ratio in the pots without strawberry for the growing media blends at the initial, intermediate and final sampling. Values are averages of 6 replicates (3 for experiment I and 3 for experiment II). Blends: pure peat and peat mixed with miscanthus straw without (MS) or with extrusion (MSEX), and with *Trichoderma* pre-inoculation (TRI). Different letters indicate significantly differences according to the Tukey HSD post-hoc test.*:0.05 and **: 0.01 significance for the factors: experiment, mixture and period tested in 3-way ANOVA (DM: dry matter, NS: not significant).**

| **Time** | **Mixture** | **pH-H2O** | **OM** | **C/N** | **Total N** | **N immob** |
| --- | --- | --- | --- | --- | --- | --- |
|  |  | **-** | **%/DM** | **-** | **%/DM** | **%** |
| Initial | Peat | 6.5 | 94 | 61 | 0.82 | 4 |
|  | MS | 6.4 | 93.6 | 68 | 0.73 | 9 |
|  | MSEX | 6.3 | 94.4 | 70 | 0.72 | 1 |
|  | MSEXTRI | 6.6 | 93.6 | 67 | 0.76 | 4 |
|  | average | 6.5 | 93.9 b | 67c | 0.76 a | 5 a |
| Intermediate | Peat | 6.4 | 93.1 | 56 | 0.93 | 15 |
|  | MS | 6.7 | 92.7 | 61 | 0.85 | 21 |
|  | MSEX | 6.5 | 94,0 | 58 | 0.87 | 20 |
|  | MSEXTRI | 6.4 | 93.2 | 56 | 0.94 | 28 |
|  | average | 6.5 | 93.3 b | 58 b | 0.90 b | 21 b |
| End | Peat | 6.3 | 89.7 | 50 | 1.01 | 24 |
|  | MS | 6.5 | 90.5 | 49 | 1.01 | 31 |
|  | MSEX | 6.6 | 92.1 | 54 | 0.96 | 29 |
|  | MSEXTRI | 6.2 | 92,0 | 48 | 1.07 | 36 |
|  | average | 6.4 | 91.1 a | 50 a | 1.01 c | 30 c |
| Experiment |  | * | NS | * | NS | NS |
| Mixture |  | NS | * | * | * | NS |
| Time |  | NS | ** | ** | ** | ** |
