# Supplementary material for: Trichoderma-Inoculated Miscanthus Straw Can Replace Peat in Strawberry Cultivation, with Beneficial Effects on Disease Control
Source: Front Plant Sci. 2018 Feb 21;9:213. doi: 10.3389/fpls.2018.00213 (PMC5826379; doi:10.3389/fpls.2018.00213)
Supplement: Supplementary file 4 [file Table4.DOCX]

**Table S4. Initial chemical characteristics of the tested growing media: pure peat and peat mixed with miscanthus straw without (MS) or with extrusion (MSEX), and with *Trichoderma* pre-inoculation (TRI), and with or without Trianum® spores (TRIspores) used in experiment 1 and 2. Values in bold are in (not underlined) or above (underlined) the optimal range for use as growing medium according to De Tender et al. (2016). Results are based on water extractable pH, electrical conductivity (EC), mineral N (NH4-N + NO3-N), P, SO4, and ammonium acetate (AmAc) extractable P, K, Ca and Mg (OM: organic matter, DM: dry matter, Nmin: mineral N, NA: not assessed). Values are averages for three replicates per treatment and per experiment.**

| **Mixture** | **Experiment** | **pH-H_2_O** | **EC** | **Nmin (H_2_O)** | **P (H_2_O)** | **SO_4_ (H_2_O)** | **P-AmAc** | **K-AmAc** | **Ca-AmAc** | **Mg-AmAc** | **OM** | **C/N** |
| --- | --- | --- | --- | --- | --- | --- | --- | --- | --- | --- | --- | --- |
|  |  | **-** | **µS/cm** | **mg/L substrate** | | | | | | | **%/DM** | **-** |
| Peat | I | **6.5** | 93 | 25.1 | 11 | 67 | 15.4 | 78 | **762** | **159** | 93.6 | 65.9 |
| Peat | II | **6.5** | 123 | 35.6 | 14.5 | 75 | 21.8 | 64 | **853** | **158** | 94.4 | 56.2 |
| Peat + TRIspores | II | **6.4** | 147 | 49.9 | 17.3 | 99 | 27.6 | 78 | **854** | **159** | 92.2 | 64.3 |
| MS | I | **6.2** | 116 | 19.8 | 18.2 | 83 | 19.7 | 112 | **740** | **150** | 93.7 | 67.3 |
| MS | II | **6.7** | 135 | 29.1 | 14.9 | 77 | 23.4 | **164** | **693** | 127 | 93.6 | 68.3 |
| MS + TRIspores | II | **6.4** | 125 | 25.0 | 13.2 | 74 | 22.6 | **158** | **683** | 127 | 93.3 | 66.6 |
| MSEX | I | **6.0** | 71 | 4.5 | 11 | 52 | 15.8 | 88 | **609** | 120 | 94.3 | 75.3 |
| MSEX | II | **6.6** | 138 | 20.3 | 15.9 | 90 | 16.9 | 93 | **556** | 102 | 94.5 | 65.6 |
| MSEX + TRIspores | II | **6.3** | 126 | 23.7 | 13.3 | 86 | 19.5 | 106 | **641** | 125 | 94.1 | 61.4 |
| MSEXTRI | I | **6.5** | 109 | 27.2 | 17.2 | 73 | 28 | 108 | **684** | 136 | 93.9 | 68.2 |
| MSEXTRI | II | **6.6** | 129 | 28.3 | 18.6 | 67 | 28.5 | 110 | **698** | 116 | 93.4 | 66 |
| MSEXTRI + TRIspores | II | **6.4** | 155 | 42.4 | 20.3 | 92 | 27.8 | 116 | **738** | 125 | 93.8 | 63.8 |
| Lower limit optimal range |  | 3.8 | 200 | 60 |  | 0 | 30 | 150 | 325 | 150 |  |  |
| Upper limit optimal range |  | 6 | 400 | 140 |  | 100 | 70 | 360 | 2100 | 300 |  |  |
