# Supplementary material for: Trichoderma-Inoculated Miscanthus Straw Can Replace Peat in Strawberry Cultivation, with Beneficial Effects on Disease Control
Source: Front Plant Sci. 2018 Feb 21;9:213. doi: 10.3389/fpls.2018.00213 (PMC5826379; doi:10.3389/fpls.2018.00213)
Supplement: Supplementary file 5 [file Table5.docx]

**Table S5. Nutrients measured in the fertigation water at two time points during experiment I and II.**

| Nutrient (mg/L) | Experiment I | Experiment II |
| --- | --- | --- |
| Fe | 0.16 ± 0.05 | 0.44 ± 0 |
| Al | 0.1 ± 0.02 | 0.07 ± 0 |
| Cu | 0.27 ± 0.08 | 0.30 ± 0.07 |
| Zn | 0.22 ± 0 | 0.40 ± 0.003 |
| Mn | 0.18 ± 0.02 | 0.21 ± 0.02 |
| Mg | 8.29 ± 0.16 | 9.92 ± 1.16 |
| Ca | 50.94 ± 3.03 | 41.53 ± 1.97 |
| K | 40.85 ± 3.91 | 45.6 ± 6.07 |
| Na | 4.46 ± 0.03 | 4.95 ± 0.84 |
| NO_3_ | 200.6 0± 8.63 | 200.45 ± 15.59 |
| NH_4_ | 30.11 ± 12.10 | 34.08 ± 3.56 |
| PO_4_ | 22.90 ± 2.40 | 28.30 ± 5.59 |
| SO_4_ | 143.35 ± 4.60 | 142.15 ± 16.16 |
