# Supplementary material for: Trichoderma-Inoculated Miscanthus Straw Can Replace Peat in Strawberry Cultivation, with Beneficial Effects on Disease Control
Source: Front Plant Sci. 2018 Feb 21;9:213. doi: 10.3389/fpls.2018.00213 (PMC5826379; doi:10.3389/fpls.2018.00213)
Supplement: Supplementary file 6 [file Table6.docx]

**Table S6. Statistical significant fungal genera (p<0.05) between at least two mixtures in either experiment I or experiment II.** Table headings represent either miscanthus straw without (MS) or with extrusion (MSEX), and with *Trichoderma* pre-inoculation (TRI), and peat, and with or without Trianum® spores (TRIspores). Values represent the mean relative abundance (%) of the genera ± standard error (number of replicates = 5).

| **Phylum** | **Genus** | **Experiment 1** | | | | | **Experiment 2** | | | | | |  |
| --- | --- | --- | --- | --- | --- | --- | --- | --- | --- | --- | --- | --- | --- |
|  |  | Peat | MS | MSEX | MSEXTRI | Peat | | MS | MSEX | MSEXTRI | Peat + TRIspores | MSEX + TRIspores | |
| Ascomycota | *Acremonium* | 0.17±0.06 (b) | 0.03±0.01 (ab) | 0.01±0.01 (a) | 0.02±0.01 (ab) | 0.03±0.02 (a) | | 0.00±0.00 (a) | 0.00±0.00 (a) | 0.00±0.00 (a) | 0.01±0.01 (a) | 0.00±0.00 (a) | |
|  | *Alternaria* | 0.25±0.11 (b) | 0.02±0.01 (a) | 0.08±0.04 (ab) | 0.02±0.01 (a) | 0.01±0.01 (a) | | 0.02±0.01 (a) | 0.00±0.00 (a) | 0.02±0.02 (a) | 0.04±0.02 (a) | 0.02±0.01 (a) | |
|  | *Arnium* | 0.01±0.00 (a) | 0.04±0.04 (ab) | 0.17±0.03 (b) | 0.01±0.01 (a) | 0.00±0.00 (a) | | 0.00±0.00 (a) | 0.00±0.00 (a) | 0.00±0.00 (a) | 0.00±0.00 (a) | 0.00±0.00 (a) | |
|  | *Arthrobotrys* | 0.03±0.02 (a) | 0.21±0.10 (b) | 0.02±0.01 (a) | 0.01±0.00 (a) | 0.05±0.01 (b) | | 0.00±0.00 (a) | 0.00±0.00 (a) | 0.00±0.00 (a) | 0.01±0.01 (a) | 0.00±0.00 (a) | |
|  | *Blastobotrys* | 0.20±0.08 (b) | 0.10±0.04 (ab) | 0.05±0.02 (a) | 0.03±0.01 (a) | 0.00±0.00 (a) | | 0.00±0.00 (a) | 0.00±0.00 (a) | 0.00±0.00 (a) | 0.00±0.00 (a) | 0.00±0.00 (a) | |
|  | *Chaetomium^*^* | 0.22±0.12 (a) | 1.31±0.57 (b) | 0.07±0.02 (a) | 0.35±0.02 (ab) | 0.15±0.05 (a) | | 0.76±0.29 (b) | 0.11±0.01 (a) | 0.12±0.04 (a) | 0.12±0.04 (a) | 0.15±0.09 (a) | |
|  | *Cladorrhinum* | 0.06±0.06 (a) | 0.00±0.00 (a) | 0.00±0.00 (a) | 0.86±0.68 (b) | 0.00±0.00 (a) | | 0.02±0.02 (a) | 0.02±0.02 (a) | 0.01±0.01 (a) | 0.00±0.00 (a) | 0.00±0.00 (a) | |
|  | *Cladosporium^*^* | 5.23±1.21 (b) | 0.52±0.26 (a) | 0.67±0.12 (a) | 0.29±0.03 (a) | 1.55±0.79 (b) | | 0.28±0.17 (a) | 0.13±0.03 (a) | 0.44±0.16 (a) | 0.85±0.19 (ab) | 0.19±0.05 (a) | |
|  | *Exophiala* | 0.02±0.01 (b) | 0.02±0.01 (b) | 0.00±0.00 (a) | 0.00±0.00 (a) | 0.18±0.05 (b) | | 0.06±0.02 (ab) | 0.02±0.01 (a) | 0.11±0.06 (ab) | 0.13±0.04 (ab) | 0.02±0.01 (a) | |
|  | *Fusarium* | 0.09±0.05 (b) | 0.02±0.01 (a) | 0.02±0.01 (a) | 0.06±0.02 (ab) | 0.55±0.40 (b) | | 0.02±0.01 (a) | 0.02±0.00 (a) | 0.04±0.02 (a) | 0.36±0.09 (b) | 0.01±0.00 (a) | |
|  | *Geomyces^*^* | 0.03±0.01 (a) | 0.06±0.02 (b) | 0.57±0.06 (b) | 0.00±0.00 (a) | 1.04±0.56 (b) | | 0.51±0.03 (ab) | 0.34±0.18 (a) | 0.39±0.35 (a) | 0.96±0.21 (b) | 0.46±0.11 (a) | |
|  | *Humicola* | 4.07±1.16 (a) | 14.48±4.23 (b) | 1.09±0.21 (a) | 54.01±1.84 (c) | 2.53±0.89 (a) | | 28.81±5.30 (b) | 38.69±2.31 (b) | 47.53±12.74 (c) | 7.07±5.02 (a) | 30.44±1.24 (b) | |
|  | *Hyaloscypha* | 0.04±0.01 (a) | 0.00±0.00 (a) | 0.01±0.00 (a) | 0.00±0.00 (a) | 0.20±0.17 (b) | | 0.00±0.00 (a) | 0.00±0.00 (a) | 0.00±0.00 (a) | 0.10±0.01 (ab) | 0.00±0.00 (a) | |
|  | *Hyphopichia* | 0.02±0.02 (a) | 0.00±0.00 (a) | 0.00±0.00 (a) | 0.02±0.01 (a) | 0.08±0.07 (b) | | 0.01±0.01 (ab) | 0.00±0.00 (a) | 0.03±0.00 (ab) | 0.08±0.05 (b) | 0.02±0.01 (ab) | |
|  | *Hypocrea* | 0.06±0.01 (a) | 0.86±0.40 (b) | 0.01±0.01 (a) | 0.01±0.01 (a) | 0.00±0.00 (a) | | 0.00±0.00 (a) | 0.00±0.00 (a) | 0.00±0.00 (a) | 0.00±0.00 (a) | 0.00±0.00 (a) | |
|  | *Ilyonectria^*^* | 1.15±0.64 (b) | 0.04±0.02 (a) | 0.06±0.04 (a) | 0.02±0.01 (a) | 0.11±0.05 (b) | | 0.04±0.04 (ab) | 0.00±0.00 (a) | 0.01±0.00 (a) | 0.04±0.01 (ab) | 0.01±0.01 (a) | |
|  | *Lecanicillium^*^* | 1.12±0.23 (b) | 0.06±0.03 (a) | 0.24±0.14 (ab) | 0.06±0.02 (a) | 0.53±0.24 (b) | | 0.06±0.01 (a) | 0.08±0.02 (a) | 0.14±0.06 (a) | 0.82±0.27 (b) | 0.14±0.07 (a) | |
|  | *Lecythophora* | 0.01±0.00 (a) | 0.02±0.02 (a) | 0.11±0.01 (b) | 0.00±0.00 (a) | 0.00±0.00 (a) | | 0.00±0.00 (a) | 0.00±0.00 (a) | 0.00±0.00 (a) | 0.00±0.00 (a) | 0.01±0.01 (a) | |
|  | *Meliniomyces^*^* | 0.11±0.08 (b) | 0.01±0.01 (a) | 0.02±0.01 (ab) | 0.01±0.01 (a) | 1.02±0.29 (b) | | 0.08±0.06 (a) | 0.06±0.04 (a) | 0.21±0.09 (a) | 0.22±0.08 (a) | 0.13±0.08 (a) | |
|  | *Oidiodendron* | 0.33±0.08 (b) | 0.04±0.01 (a) | 0.05±0.01 (a) | 0.03±0.01 (a) | 0.56±0.11 (b) | | 0.05±0.01 (a) | 0.06±0.02 (a) | 0.11±0.06 (a) | 0.39±0.12 (b) | 0.09±0.04 (a) | |
|  | *Ophiostoma^*^* | 0.13±0.08 (b) | 0.03±0.02 (ab) | 0.01±0.00 (a) | 0.08±0.02 (ab) | 2.57±0.67 (a) | | 2.39±0.66 (a) | 1.41±0.23 (a) | 3.39±0.52 (a) | 2.72±0.24 (a) | 2.45±0.35 (a) | |
|  | *Parap-haeosphaeria^*^* | 0.02±0.02 (a) | 0.17±0.08 (a) | 0.04±0.01 (a) | 0.02±0.01 (a) | 0.05±0.02 (a) | | 3.29±0.91 (c) | 0.80±0.15 (b) | 0.54±0.35 (b) | 0.16±0.11 (a) | 0.97±0.71 (b) | |
|  | *Penicillium* | 0.60±0.12 (b) | 0.11±0.03 (a) | 0.11±0.03 (a) | 0.09±0.02 (a) | 0.63±0.18 (b) | | 0.14±0.01 (a) | 0.22±0.02 (a) | 0.19±0.14 (a) | 0.58±0.13 (b) | 0.16±0.03 (a) | |
|  | *Pestalotiopsis* | 0.15±0.08 (b) | 0.01±0.01 (a) | 0.01±0.01 (a) | 0.01±0.01 (a) | 0.24±0.07 (b) | | 0.04±0.02 (ab) | 0.01±0.00 (a) | 0.04±0.02 (ab) | 0.12±0.05 (ab) | 0.07±0.05 (ab) | |
|  | *Peyronellaea* | 0.51±0.13 (b) | 0.05±0.02 (a) | 0.03±0.00 (a) | 0.05±0.04 (a) | 0.44±0.32 (b) | | 0.04±0.01 (a) | 0.05±0.02 (a) | 0.06±0.02 (a) | 0.52±0.26 (b) | 0.07±0.01 (a) | |
|  | *Pezicula* | 0.00±0.00 (a) | 0.00±0.00 (a) | 0.00±0.00 (a) | 0.00±0.00 (a) | 0.44±0.02 (c) | | 0.11±0.03 (b) | 0.02±0.01 (a) | 0.36±0.30 (ab) | 0.21±0.08 (abc) | 0.13±0.05 (ab) | |
|  | *Phialocephala* | 0.20±0.12 (b) | 0.01±0.01 (ab) | 0.01±0.01 (ab) | 0.00±0.00 (a) | 0.00±0.00 (a) | | 0.00±0.00 (a) | 0.00±0.00 (a) | 0.01±0.00 (a) | 0.00±0.00 (a) | 0.00±0.00 (a) | |
|  | *Pilidium^*^* | 0.00±0.00 (a) | 0.00±0.00 (a) | 0.00±0.00 (a) | 0.00±0.00 (a) | 3.00±1.08 (c) | | 1.59±0.47 (bc) | 0.24±0.11 (a) | 1.38±0.66 (b) | 2.51±1.04 (c) | 0.84±0.44 (ab) | |
|  | *Podospora^*^* | 0.02±0.01 (a) | 0.57±0.26 (b) | 0.06±0.04 (ab) | 0.29±0.26 (ab) | 0.07±0.04 (a) | | 0.75±0.29 (b) | 2.14±1.25 (b) | 0.50±0.34 (b) | 0.01±0.81 (a) | 0.55±0.22 (b) | |
|  | *Pseudo-gymnoascus^*^* | 0.15±0.03 (b) | 0.01±0.00 (a) | 0.12±0.01 (b) | 0.01±0.01 (a) | 0.59±0.16 (b) | | 1.07±0.10 (b) | 0.74±0.13 (ab) | 0.24±0.11 (a) | 0.69±0.20 (b) | 0.75±0.26 (b) | |
|  | *Simplicillium* | 0.43±0.16 (b) | 0.03±0.02 (a) | 0.09±0.02 (ab) | 0.10±0.02 (ab) | 0.18±0.11 (b) | | 0.02±0.00 (a) | 0.02±0.01 (a) | 0.07±0.03 (ab) | 0.14±0.05 (b) | 0.02±0.01 (a) | |
|  | *Sordaria* | 0.01±0.01 (a) | 0.15±0.12 (b) | 0.46±0.13 (b) | 0.00±0.00 (a) | 0.02±0.01 (b) | | 0.00±0.00 (a) | 0.00±0.00 (a) | 0.00±0.00 (a) | 0.00±0.00 (a) | 0.00±0.00 (a) | |
|  | *Sporothrix^*^* | 0.15±0.03 (a) | 0.92±0.39 (b) | 1.19±0.18 (b) | 0.63±0.15 (b) | 0.14±0.02 (a) | | 0.86±0.19 (b) | 0.86±0.19 (b) | 0.93±0.34 (b) | 0.53±0.27 (ab) | 1.78±0.30 (b) | |
|  | *Stachybotrys* | 0.01±0.00 (a) | 0.02±0.02 (a) | 0.42±0.33 (b) | 0.00±0.00 (a) | 0.05±0.03 (a) | | 0.00±0.00 (a) | 0.01±0.01 (a) | 0.00±0.00 (a) | 0.02±0.01 (a) | 0.00±0.00 (a) | |
|  | *Talaromyces* | 0.06±0.02 (b) | 0.01±0.00 (a) | 0.01±0.01 (a) | 0.00±0.00 (a) | 0.37±0.11 (b) | | 0.04±0.02 (a) | 0.02±0.00 (a) | 0.06±0.03 (a) | 0.16±0.08 (ab) | 0.05±0.04 (a) | |
|  | *Tetracladium^*^* | 1.38±0.90 (b) | 0.04±0.02 (a) | 0.06±0.02 (a) | 0.06±0.03 (a) | 0.00±0.00 (a) | | 0.00±0.00 (a) | 00±0.00 (a) | 0.00±0.00 (a) | 0.00±0.00 (a) | 0.00±0.00 (a) | |
|  |  | Peat | MS | MSEX | MSEXTRI | Peat | | MS | MSEX | MSEXTRI | Peat + TRIspores | MSEX + TRIspores | |
| Ascomycota | *Trichoderma* | 0.07±0.04 (b) | 0.10±0.04 (b) | 0.02±0.01 (ab) | 0.00±0.00 (a) | 0.24±0.07 (b) | | 0.14±0.01 (ab) | 0.19±0.03 (ab) | 0.32±0.30 (a) | 0.88±0.77 (c) | 0.12±0.03 (ab) | |
|  | *Venturia* | 0.89±0.17 (b) | 0.16±0.05 (a) | 0.28±0.02 (a) | 0.28±0.07 (a) | 0.00±0.00 (a) | | 0.00±0.00 (a) | 0.00±0.00 (a) | 0.00±0.00 (a) | 0.00±0.00 (a) | 0.00±0.00 (a) | |
|  | *Zopfiella^*^* | 0.52±0.18 (a) | 1.63±0.74 (ab) | 8.68±0.85 (b) | 1.73±0.53 (ab) | 0.09±0.05 (a) | | 0.05±0.05 (a) | 0.19±0.10 (a) | 0.10±0.08 (a) | 0.04±0.01 (a) | 0.04±0.03 (a) | |
| Basidiomycota | *Agrocybe* | 0.15±0.05 (a) | 0.01±0.00 (b) | 0.03±0.01 (b) | 0.04±0.02 (b) | 0.00±0.00 (a) | | 0.00±0.00 (a) | 0.00±0.00 (a) | 0.00±0.00 (a) | 0.00±0.00 (a) | 0.00±0.00 (a) | |
|  | *Basidio-*  *dendron* | 0.26±0.12 (b) | 0.01±0.01 (a) | 0.04±0.04 (ab) | 0.01±0.00 (a) | 0.00±0.00 (a) | | 0.00±0.00 (a) | 0.00±0.00 (a) | 0.00±0.00 (a) | 0.00±0.00 (a) | 0.00±0.00 (a) | |
|  | *Clitopilus* | 0.51±0.35 (b) | 0.03±0.01 (a) | 0.01±0.00 (a) | 0.00±0.01 (a) | 0.05±0.05 (a) | | 0.00±0.00 (a) | 0.00±0.00 (a) | 0.00±0.00 (a) | 0.00±0.00 (a) | 0.00±0.00 (a) | |
|  | *Cryptococcus^*^* | 1.69±0.24 (b) | 0.21±0.06 (a) | 0.56±0.08 (ab) | 0.17±0.02 (a) | 5.88±1.61 (b) | | 2.39±0.92 (ab) | 1.47±0.10 (a) | 1.85±0.75 (a) | 3.23±0.63 (b) | 1.52±0.27 (a) | |
|  | *Cystobasidium* | 0.54±0.07 (b) | 0.04±0.02 (a) | 0.15±0.03 (a) | 0.04±0.02 (a) | 0.11±0.03 (b) | | 0.04±0.01 (ab) | 0.01±0.00 (a) | 0.08±0.02 (ab) | 0.02±0.01 (ab) | 0.06±0.05 (ab) | |
|  | *Flagelloscypha* | 0.34±0.30 (b) | 0.01±0.01 (ab) | 0.00±0.00 (a) | 0.01±0.01 (ab) | 0.55±0.46 (ab) | | 0.09±0.02 (a) | 0.07±0.04 (a) | 0.01±0.01 (a) | 0.50±0.16 (b) | 0.11±0.06 (a) | |
|  | *Guehomyces* | 0.01±0.01 (b) | 0.01±0.00 (b) | 0.01±0.01 (b) | 0.00±0.00 (a) | 0.03±0.02 (a) | | 0.05±0.01 (a) | 0.22±0.04 (b) | 0.02±0.02 (a) | 0.07±0.03 (a) | 0.18±0.06 (ab) | |
|  | *Leucosporidiella* | 0.00±0.00 (a) | 0.00±0.00 (a) | 0.12±0.02 (b) | 0.00±0.00 (a) | 0.31±0.12 (b) | | 0.08±0.03 (ab) | 0.02±0.01 (a) | 0.10±0.04 (a) | 0.19±0.04 (ab) | 0.04±0.02 (a) | |
|  | *Meira* | 0.06±0.03 (a) | 0.01±0.01 (a) | 0.02±0.01 (a) | 0.01±0.00 (a) | 0.35±0.20 (b) | | 0.07±0.03 (ab) | 0.04±0.02 (a) | 0.07±0.03 (ab) | 0.22±0.04 (b) | 0.06±0.03 (ab) | |
|  | *Occultifur* | 0.08±0.03 (b) | 0.00±0.00 (a) | 0.03±0.01 (ab) | 0.00±0.00 (a) | 0.24±0.15 (b) | | 0.08±0.06 (ab) | 0.01±0.00 (a) | 0.03±0.01 (a) | 0.06±0.02 (ab) | 0.00±0.00 (a) | |
|  | *Pluteus* | 0.00±0.00 (a) | 0.00±0.00 (a) | 0.11±0.02 (b) | 0.00±0.00 (a) | 0.00±0.00 (a) | | 0.00±0.00 (a) | 0.00±0.00 (a) | 0.11±0.10 (b) | 0.00±0.00 (a) | 0.00±0.00 (a) | |
|  | *Rhodotorula* | 0.55±0.12 (b) | 0.08±0.04 (a) | 0.07±0.01 (a) | 0.03±0.01 (a) | 0.27±0.02 (b) | | 0.07±0.02 (a) | 0.04±0.01 (a) | 0.17±0.07 (ab) | 0.40±0.16 (b) | 0.08±0.05 (a) | |
|  | *Trichosporon^*^* | 3.63±1.85 (b) | 0.22±0.09 (a) | 0.44±0.29 (a) | 0.12±0.04 (a) | 9.63±2.20 (a) | | 11.16±2.52 (a) | 5.81±1.03 (a) | 10.31±9.33 (b) | 17.08±5.18 (b) | 10.44±2.29 (a) | |
| Chytridiomycota | *Kochiomyces* | 0.18±0.10 (b) | 0.00±0.00 (a) | 0.00±0.00 (a) | 0.00±0.00 (a) | 0.10±0.05 (a) | | 0.04±0.03 (a) | 0.00±0.00 (a) | 0.03±0.02 (a) | 0.05±0.04 (a) | 0.03±0.02 (a) | |
|  | *Olpidium* | 0.29±0.12 (a) | 0.12±0.08 (a) | 0.17±0.04 (a) | 0.08±0.02 (a) | 0.77±0.67 (b) | | 0.11±0.06 (ab) | 0.03±0.00 (a) | 0.06±0.03 (a) | 0.25±0.06 (b) | 0.03±0.02 (a) | |
| Glomeromycota | *Claroideoglomus* | 0.02±0.01 (a) | 0.09±0.02 (b) | 0.00±0.00 (a) | 0.00±0.00 (a) | 0.00±0.00 (a) | | 0.00±0.00 (a) | 0.00±0.00 (a) | 0.00±0.00 (a) | 0.00±0.00 (a) | 0.00±0.00 (a) | |
|  | *Rhizophagus* | 0.79±0.50 (b) | 0.03±0.01 (a) | 0.11±0.03 (a) | 0.07±0.03 (a) | 0.03±0.02 (a) | | 0.12±0.05 (a) | 0.08±0.06 (a) | 0.06±0.04 (a) | 0.29±0.24 (a) | 0.07±0.04 (a) | |
| Zygomycota | *Mortierella^*^* | 14.61±3.65 (b) | 4.12±1.68 (ab) | 1.44±0.63 (a) | 0.54±0.23 (a) | 2.90±0.77 (a) | | 5.81±0.84 (b) | 14.43±1.29 (b) | 1.58±0.64 (a) | 2.43±0.45 (a) | 16.71±2.24 (b) | |
|  | *Mucor* | 0.04±0.02 (a) | 0.24±0.22 (ab) | 1.24±0.20 (b) | 0.01±0.01 (a) | 0.07±0.04 (a) | | 0.61±0.08 (b) | 0.90±0.17 (b) | 0.02±0.01 (a) | 0.05±0.02 (a) | 0.53±0.07 (b) | |
|  | *Umbelopsis^*^* | 0.96±0.28 (b) | 0.21±0.07 (a) | 0.11±0.01 (a) | 0.08±0.02 (a) | 0.42±0.09 (b) | | 0.10±0.02 (a) | 0.23±0.03 (b) | 0.10±0.06 (a) | 0.24±0.06 (b) | 0.08±0.02 (a) | |

^*^Genera representing at least 1 % of the total community in at least one mixture in either experiment I or II (Represented in Table 5)
